# Bytes and bites: social media use and dietary behaviours among adolescents across 41 countries

**DOI:** 10.1038/s41390-025-04030-z

**Published:** 2025-04-07

**Authors:** Asaduzzaman Khan, Jie Feng, Veronique Chachay, Jaclyn H. Tsang, Wendy Y. Huang, Cindy H. P. Sit, Victor Minichiello

**Affiliations:** 1 School of Health and Rehabilitation Sciences, The University of Queensland, Brisbane, QLD Australia; 2 Department of Sports Science and Physical Education, The Chinese University of Hong Kong, Hong Kong, China; 3 Frazer Institute, The University of Queensland, Brisbane, QLD Australia; 4 Department of Health and Physical Education, The Education University of Hong Kong, Hong Kong, China; 5 Academy of Wellness and Human Development, Hong Kong Baptist University, Hong Kong, China; 6 Dr. Stephen Hui Research Centre for Physical Recreation and Wellness, Hong Kong Baptist University, Hong Kong, China; 7 School of Social Justice, Faculty of Creative Industries, Education and Social Justice, Queensland University of Technology, Brisbane, QLD Australia

## Abstract

**Background:**

To examine the associations of problematic and excessive social media use (SMU) with dietary behaviours of adolescents.

**Methods:**

We analysed the 2017/2018 Health Behaviour in School-aged Children survey data, involving 222,865 adolescents (51.8% girls) from 41 countries. A dietary intake score was derived using consumption of fruits, vegetables, sweets, and sugary soft-drinks. Breakfast intake was categorised as daily or non-daily. Excessive SMU assessed how often respondents had online contact through social media, and problematic SMU was assessed through symptoms of addiction.

**Results:**

Regression analyses showed that adolescents reporting problematic SMU had 54% lower odds in boys (OR 0.46; 95% CI 0.42–0.51) and 64% lower odds in girls (OR 0.36; 0.33–0.40) of reporting good dietary intake compared with poor intake. Excessive SMU was also associated with lower odds of reporting good dietary intake. Problematic SMU associated inversely with daily fruit and vegetable intake, while excessive SMU was positively associated with daily fruit and vegetable intake across sex. Both types of SMU were linked to increased intake of sweets and sugary drinks and decreased breakfast consumption.

**Conclusions:**

Problematic SMU was associated with poor dietary habits, while excessive SMU showed mixed findings. Prospective research is warranted to understand the causal mechanisms.

**Impact:**

- Problematic and excessive social media use (SMU) was associated with poor dietary habits with problematic SMU being more detrimental than excessive SMU.
- This research contributes to the literature by demonstrating that problematic and excessive SMU correlate differently with adolescent dietary habits, highlighting the need for targeted approaches to promote healthier eating.
- Adolescents should be encouraged to use social media responsibly, while social media companies should promote local fresh food options to enhance healthy dietary habits.

## Introduction

Dietary behaviours are crucial for children’s physical, cognitive, and emotional development, as well as for establishing long-term health and preventing future chronic diseases.^1,2^ Adolescence offers a unique opportunity to develop and promote healthy dietary habits, as it is a critical period characterised by the emergence of autonomy in eating behaviours and the establishment of new personal habits.^3^ Despite the well documented benefits, unhealthy dietary habits are prevalent among adolescents in both developing^4^ and developed^5^ countries. Examining data from 23 European countries, the latter study reported that each day, less than half (42.5%) of children consumed fruit, and fewer than a quarter (22.6%) consumed fresh vegetables, indicating lack of alignment with the dietary guidelines.^5^

A meta-analysis including 1.43 million young people indicated that adolescents who viewed content related to health risk behaviours were more inclined to eat unhealthy foods compared to those who did not see such content.^6^ Dietary habits of adolescents are influenced by multiple factors including personal, behavioural, and socioenvironmental factors, including the fast-changing social media environment. A systematic review found that excessive social media use (SMU) was associated with unhealthy dietary habits such as skipping breakfast, lower intake of vegetables and fruits, and higher consumption of unhealthy snacks and beverages, in children and adolescents.^7^ A systematic review reported that social media engagement or exposure to image-related content may negatively impact food choice in healthy young adults.^8^ A study conducted among Belgian adolescents aged 11–19 years found that social media exposure was associated with both positive and negative eating outcomes in adolescents.^9^ In contrast, evidence on the link between social media exposure and healthy food intake is limited. Recent research investigated the role of social media on healthy food intake and nutrition literacy among children and adolescents^7,9^ and found that SMU was linked to unhealthy dietary behaviours (e.g., lower intake of fruits and vegetables, higher rates of breakfast skipping). A recent study reported that adolescents who were frequently exposed to social media messages about healthy foods (e.g., fruits and vegetables, primarily posted by peers, celebrities, or influencers) had an increased intake of healthy foods compared to their counterparts who were not frequently exposed.^9^ The mixed and inconclusive nature of these findings may result from the diverse measures of social media exposure and dietary behaviours. For example, one study specifically examined the exposure to social media messages and food marketing;^9^ while another covered a broader range of parameters including interaction on social networking platform, food marketing, exposure to food images, and overall internet/smartphone use.^7^ The differential results show that social media can impact what users consume, which depends on various factors, such as how the platforms are designed or the use of artificial intelligence to select food-related content.

Instead of excessive use of social media, problematic SMU (i.e., addiction-like symptoms such as loss of control, tolerance, and preoccupation) may influence adolescent health and behaviours. Earlier research has observed relationships between problematic SMU, such as social media addiction, and eating behaviours among adolescents and young adults.^10,11^ However, none of the previous studies have examined the links using an SMU scale that includes a distinction between intensity and problematic use. The absence of addiction-like symptoms related to social media may contribute to the mixed findings regarding the relationships between SMU and dietary habits. Furthermore, the existing knowledge regarding the relationship between SMU and dietary behaviours primarily relies on small or unrepresentative samples and lacks comprehensive assessments of social media engagement and eating patterns. Available evidence also suggests significant sex-based differences in how adolescents interact with social media and how these interactions influence their food preferences and consumption.^12^ To address these research gaps, this study aimed to examine the relationships of problematic and excessive use of social media with measures of dietary intake and habitual breakfast consumption across sex.

## Methods

The current analysis was based on the 2017–2018 Health Behaviour in School-aged Children (HBSC) survey, which is a cross-sectional health and wellbeing survey of adolescents across European and North American countries. Participants anonymously provided self-reported data by completing a questionnaire covering various health indicators and related behaviours. Survey administrators in each country obtained ethics approval from an appropriate regulatory body and secured informed consent from both the participants and a parent or guardian. Of the 45 countries that took part in the survey, four countries did not have information on the key variables and hence, the analytical sample was based on 41 countries. Secondary analyses for this manuscript were approved by The University of Queensland Human Ethics Research Committee (2021/HE000671).

### Dietary habits

In HBSC, the participating students were asked to assess how often they consume the following food groups and beverages in a typical week: (a) fruits, (b) vegetables/salads, (c) sweets (including chocolate and candies), and (d) sugary beverages, with seven responses, ranging from “never” to “more than once a day.” Based on the current WHO recommendations,^13^ the food frequencies were dichotomised as having fruit and vegetables at least daily; and consume sugary foods and drinks less than daily. To further evaluate the overall dietary behaviour, a dietary pattern quality score was derived by grouping the intake frequencies into five categories.^14^ Healthy food items (e.g., vegetables/salads and fruits) were scored as 0: ≤1 day/week; 1: 2–4 days/week; 2: 5–6 days/week; 3: 7 days/week, and 4: >once/day. Unhealthy food items (i.e., sweets and sugary beverages) were scored as 4: ≤1 day/week; 3: 2–4 days/week; 2: 5–6 days/week; 1: 7 days/week; and 0: > once/day. A total dietary intake score was computed (range: 0–16) and categorised into three groups: poor: 0–9, medium: 10–12, and good: 13–16.^15^ To assess the habitual breakfast intake, participants were asked to report how many days they usually ate breakfast on school days and during weekends, respectively. The responses were dichotomised as having breakfast every day or not.

### Social media uses

A 4-item scale from the European Union Kids Online Survey was used to assess excessive SMU, with good validity and reliability across countries.^16^ Respondents were asked how often they have online contact through social media (e.g., Facebook, WhatsApp, Snapchat) with the following people: close friends, friends from a larger friend group, friends that they met through the internet, and others (e.g., parents, siblings, classmates, teachers). Responses ranged from 1 (never/almost never) to 5 (almost all the time throughout the day). Respondents who answered almost all the time throughout the day on at least one item were classified as excessive users (1), and the remainder as non-extensive users (0).^17^ The 9-item Social Media Disorder Scale.^16^ was used to assess the problematic SMU. Respondents were asked to indicate whether they, in the past year, regularly could not think of anything else but social media, regularly felt dissatisfied because they wanted to spend more time on social media, often felt bad when they could not use social media, failed to spend less time on social media, regularly neglected other activities because of social media, regularly had arguments with others because of their SMU, regularly lied to parents or friends about their time spent on social media, often used social media to escape from negative feelings, and had serious conflicts with parents or siblings because of their SMU. Responses were yes/no. Respondents who answered positively to at least six items were classified as problematic users, and the remainder as non-problematic users.^16^

### Control variables

A set of covariates, as being linked to the use of electronic devices among adolescents,^18^ was controlled for in the modelling. Participants reported height and weight, which were used to generate body mass index (BMI) z-scores. Participants also reported the number of days in the past week that they participated in moderate-to-vigorous physical activities for at least 60 min daily. Family-level socioeconomic status was measured using the Family Affluence Scale, which is a composite score based on household assets including number of vehicles and computers, bedroom sharing, and number of family holidays in the past year. Given the evidence that SMU patterns are different for boys and girls^19^ and associations between electronic media use and health behaviours are dependent on age,^20^ both sex and age were also controlled for in the statistical modelling.

### Statistical analyses

Proportions of adolescents reporting daily intake of fruits, vegetables, breakfast, and less-than daily (i.e., limited) consumption of sweets and sugary drinks were computed, along with proportions of adolescents reporting different amounts of dietary intake, and problematic and excessive SMU. Missing values ranged from 0.6% (age) to 22.6% (BMI) (Table 1). To minimise biases due to the missing data, we implemented multiple imputations by chained equations (MICE). The imputed descriptive statistic values closely matched the observed values.

**Table 1:** Characteristics of study sample from 41 countries, HBSC study, 2017–2018 (*n* = 222,865).

Characteristics	Boys	Girls
Total participants	109,752	113,113
Average age (SD)^a^	13.49 (1.63)	13.51 (1.64)
Average Family Affluence Scale (SD)^b^	7.27 (2.73)	7.84 (2.76)
Average BMI (SD)^c^	19.70 (3.73)	19.40 (3.52)
Prevalence of sufficient physical activity (%)^d^	22.72	14.92
Prevalence of excessive social media use (%)^e^	31.06	37.74
Prevalence of problematic social media use (%)^f^	6.73	7.99
Prevalence of daily fruit intake (%)	36.87	43.23
Prevalence of daily vegetable intake (%)	34.85	41.87
Prevalence of non-daily sweet intake (%)	77.19	73.68
Prevalence of non-daily sugary drink (%)	82.70	86.53
Prevalence of daily breakfast intake (%)	54.43	49.02
Prevalence of diet quality (%)		
Good (%)	17.68	23.20
Moderate (%)	30.57	32.77
Poor (%)	51.75	44.04

*BMI* body mass index; *SD* Standard deviation.

Percentage of missing values: ^a^age: 0.67%; ^b^family affluence scale: 9.02%; ^c^BMI: 22.62%;.

^d^physical activity: 2.21%; ^e^Excessive social media use: 7.93%;

^f^Problematic social media use: 16.81%.

Multilevel logistic regression was used to examine the associations of the binary food items (e.g., daily fruits, daily vegetables, limited sweets, and limited sugary drinks intake) and daily breakfast intake with problematic and excessive SMU for each country. Multilevel multinomial logit model was used to obtain country level estimates of the associations of the categorical dietary intake (poor as reference). The same modelling approach was implemented in the overall sample. All models were adjusted for age, sex, BMI z-scores, family affluence, and physical activity. The sex-stratified analyses were supported by significant interactions between sex and problematic and excessive SMU (*p* < 0.001). Finally, we conducted sensitivity analyses using continuous problematic and excessive SMU scores to see whether such a change would affect the association estimates. The analyses were conducted using StataSE 17 and the estimates were presented in the form of odds ratio (OR) and 95% confidence interval (CI).

## Results

**Fig. 1:**
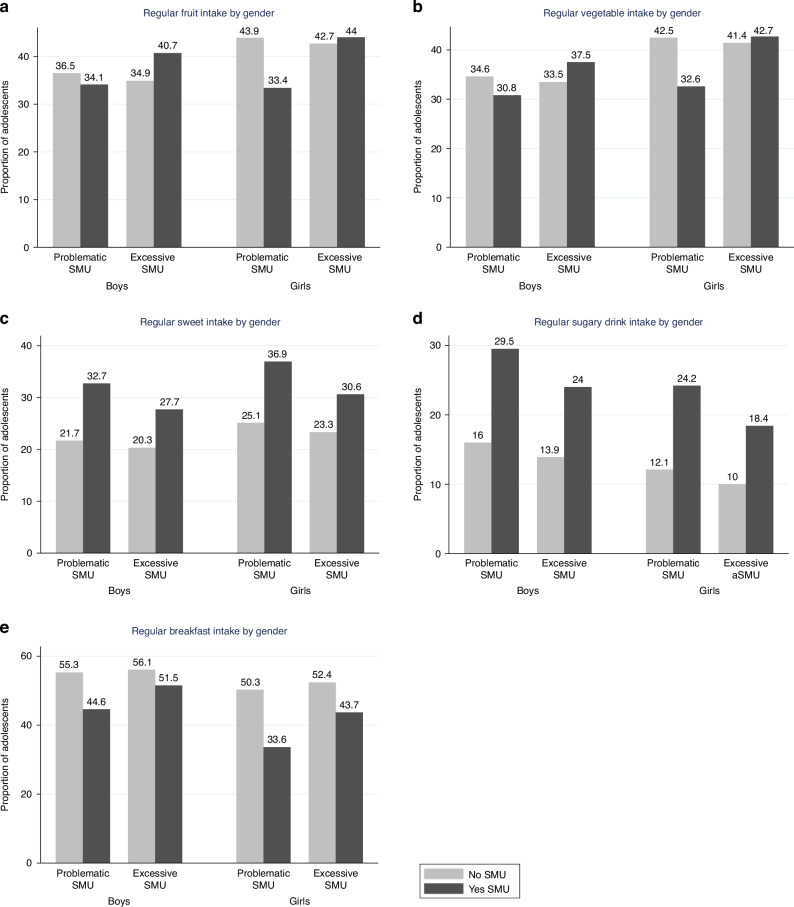
Proportions of adolescents with different dietary behaviours in relation to problematic and excessive social media use, differentiated by sex. **a** Regular intake of fruits. **b** Regular intake of vegetables. **c** Regular intake of sweets. **d** Regular intake of soft drinks. **e** Regular intake of breakfast.

Descriptive statistics of the analytical sample are presented in Table 1 (*n* = 222,865). Average age of participants was 13.50 (SD 1.63) years and 50.8% were girls. Excessive SMU was more prevalent among girls than boys (37.7% vs 31.1%) and problematic SMU was slightly higher in girls than boys (8.0% vs 6.7%). Good dietary intake was more prevalent in girls (23.2%) than boys (17.7%); while poor dietary intake was more common in boys (51.8%) than girls (44.0%). Figure 1 presents the proportions of adolescents with good dietary behaviours for problematic and excessive SMU by sex. For example, the proportion of boys consuming fruits daily was 43.9% among those non-problematic SMU, compared to 33.4% among those with problematic SMU. In contrast, the proportion of girls consuming fruits daily was 34.9% among those with non-excessive SMU, compared to 40.1% among those with excessive SMU.

Country level estimates of associations between excessive and problematic SMU and dietary intake categories are presented in Appendix A. Problematic SMU was inversely associated with good dietary intake in 25 countries in boys and in 34 countries in girls. Country level evidence on links between excessive SMU and dietary intake was mixed with 17 countries demonstrating inverse associations in girls and 7 countries showing inverse associations in boys. Association estimates for the overall sample are presented in Table 2. Multivariable modelling showed that adolescents who had problematic SMU had 54% lower odds in boys (OR 0.46; 95%CI 0.42–0.51) and 64% lower odds in girls (OR 0.36; 95%CI 0.33–0.40) of reporting good dietary intake compared to poor intake. Inverse associations were also observed when moderate dietary intake was compared with poor intake across sex. On the other hand, excessive SMU was associated with lower odds of reporting good dietary intake when compared with poor intake with no apparent sex differences (boys: OR 0.80; 95%CI 0.77–0.83; girls: OR 0.84; 95%CI 0.80–0.87).

**Table 2:** Association estimates^a^ of problematic and excessive social media use (SMU) with *diet quality* in adolescents from 41 countries, HBSC 2017/2018

	Boys		Girls	
	Good vs Poor^b^	Moderate vs Poor^b^	Good vs Poor^b^	Moderate vs Poor^b^
	aOR (95% CI)	aOR (95% CI)	aOR (95% CI)	aOR (95% CI)
Problematic SMU				
No^b^	1.00	1.00	1.00	1.00
Yes	0.46 (0.42, 0.51)	0.56 (0.52, 0.61)	0.36 (0.33, 0.40)	0.54 (0.51, 0.58)
Excessive SMU				
No^b^	1.00	1.00	1.00	1.00
Yes	0.80 (0.77, 0.83)	0.94 (0.89, 0.98)	0.84 (0.80, 0.87)	0.80 (0.78, 0.83)

*aOR* adjusted odds ratio, *CI* Confidence interval.

^a^adjusted for age, sex, BMI z-scores, family affluence, and physical activity.

^b^reference category.

Table 3 presents the association estimates of dichotomised dietary behaviours with SMU (Table 3). Adolescents reporting problematic SMU had lower odds of reporting daily intake of vegetables with lower effect observed in boys than girls (boys: OR 0.88; 95%CI 0.83–0.95; girls: OR 0.69; 95%CI 0.65–0.73). However, adolescents reporting excessive SMU had higher odds of reporting daily intake of fruits across sex (boys: OR 1.27; 95%CI 1.23–1.32; girls: OR 1.14; 95%CI 1.10–1.18). Boys with problematic SMU had 113% higher odds (OR 2.13, 95%CI 1.96–2.33) and girls with problematic SMU had 108% higher odds (OR 2.08, 95%CI 1.92–2.27) of reporting daily intake of sugary drinks. Boys with problematic SMU had 29% lower odds (OR 0.71, 95%CI 0.66–0.76), and girls with problematic SMU had 40% lower odds (OR 0.60, 95%CI 0.56–0.64), of reporting daily breakfast intake.

**Table 3:** Association estimates^a^ of problematic and excessive social media use (SMU) with daily food and breakfast intake in adolescents from 41 countries, HBSC 2017/2018

	Boys	Girls
	aOR (95% CI)	aOR (95% CI)
*Daily fruit intake*		
Problematic SMU		
No^b^	1.00	1.00
Yes	0.88 (0.83, 0.95)	0.69 (0.65, 0.73)
Excessive SMU		
No^b^	1.00	1.00
Yes	1.27 (1.23, 1.32)	1.14 (1.10, 1.18)
*Daily vegetable intake*		
Problematic SMU		
No^b^	1.00	1.00
Yes	0.86 (0.81, 0.93)	0.71 (0.66, 0.75)
Excessive SMU		
No^b^	1.00	1.00
Yes	1.18 (1.14, 1.22)	1.09 (1.06, 1.13)
*Daily consumption of sweets*		
Problematic SMU		
No^b^	1.00	1.00
Yes	1.69 (1.56, 1.82)	1.72 (1.61, 1.82)
Excessive SMU		
No^b^	1.00	1.00
Yes	1.49 (1.43, 1.56)	1.43 (1.37, 1.47)
*Daily consumption of sugary drinks*		
Problematic SMU		
No^b^	1.00	1.00
Yes	2.13 (1.96, 2.33)	2.08 (1.92, 2.27)
Excessive SMU		
No^b^	1.00	1.00
Yes	1.92 (1.85, 2.04)	2.04 (1.92, 2.13)
*Daily intake of breakfast*		
Problematic SMU		
No^b^	1.00	1.00
Yes	0.71 (0.66, 0.76)	0.60 (0.56, 0.64)
Excessive SMU		
No^b^	1.00	1.00
Yes	0.83 (0.80, 0.86)	0.76 (0.73, 0.79)

*aOR* adjusted odds ratio, *CI* Confidence interval.

^a^adjusted for age, sex, BMI z-scores, family affluence, and physical activity.

^b^reference category.

Finally, sensitivity analyses showed that problematic SMU scale scores (continuous) were inversely associated with reporting of good dietary intake, compared to poor intake, across sex (boys: OR 0.84, 95%CI 0.83–0.85; Girls: OR 0.80, 95%CI 0.79–0.81). Similarly, excessive SMU scores were inversely associated with reporting of good dietary intake, compared to poor intake, across sex (boys: OR 0.93, 95%CI 0.91–0.95; girls: OR 0.92, 95%CI 0.90–0.94).

## Discussion

Using nationally representative data from 41 countries, this study is the first to examine the relationships between problematic and excessive SMU and dietary behaviours across sex in adolescents. Those with problematic or excessive SMU had lower odds of reporting good dietary intake and daily breakfast intake. The findings suggest that problematic SMU was inversely associated with daily fruit and vegetable intake, while excessive SMU was positively associated with these eating behaviours. Both problematic and excessive SMU were positively associated with daily intake of sweets and sugary drink.

Regarding overall dietary intake, both problematic and excessive SMU were inversely associated with good dietary intake across sex, which is well aligned with previous studies.^21,22^ There are several potential explanations for these observed relationships. Firstly, the eating behaviours of adolescents may be influenced by their peers.^23^ Adolescents share and interact with unhealthy food content on social media, and spend more time looking at unhealthy food advertisements posted by peers compared to those posted by companies or celebrities.^24^ Secondly, the intake of less healthy food items may be contributed by the marketing of unhealthy beverages and snacks targeting children and adolescents on social media platforms.^25^ A previous study has found that unhealthy food advertisements are more prevalent than healthy food promotions on social media, which can lead to poorer dietary behaviours in children and adolescents.^26^

An inverse association was found between problematic SMU and daily intake of fruits and vegetables. This aligns with findings from a large study conducted in the Netherlands, which reported that problematic SMU was linked to poorer eating habits (e.g., consuming less fruits and vegetables).^27^ This inverse relationship could be attributed to mental health challenges associated with problematic SMU. Problematic SMU has been consistently linked to a higher risk of lower well-being,^17^ poorer emotion regulation,^28^ and greater depressive symptoms^29^ among adolescents, while poor well-being outcomes have been found to be associated with low fruit and vegetable consumption in adolescents.^30^ However, most previous studies have solely focused on the relationship between the intensity of SMU, rather than problematic use, and fruit and vegetable intake.^7,31^ Adolescents engaged in problematic SMU may engage in mindless eating, where they eat unhealthy snacks while scrolling, often ignoring the nutritional quality of their food; they may have limited time available for meal preparation, resulting in greater consumption of convenient, processed foods instead of healthier options like fruits and vegetables. Further research is warranted to investigate the nuanced relationships between different aspects of SMU and detailed dietary habits, as well as their mediating factors, particularly to evaluate their directionality.

Our study showed that excessive SMU was positively associated with daily intake of fruits and vegetables across sex. This observed association may be explained by the established link between SMU, body image, and dietary behaviours. Specifically, SMU was found to be associated with dietary behaviours mediated by heightened appearance comparison and body dissatisfaction.^32^ One study reported that longer exposure to SMU was associated with lower intake of fruits and vegetables,^21^ while other studies found no significant relationship.^9,33^ This inconsistency in the findings may be attributed to the varying definitions and measurements of SMU across studies. For example, some authors have defined excessive SMU as near-constant online contact with friends, family, and peers through social media,^17^ while others have characterised excessive SMU in various ways, including general internet use,^21^ exposure to food messages on social media,^9^ and screen device use.^33^ However, the current secondary analysis did not include such detailed information, limiting the ability to directly compare the results. A more standardised approach to measuring and characterising SMU, including context and contents, may help reconcile such inconsistencies.

Both problematic and excessive SMU were positively associated with daily intake of sweets and sugary drinks, similar to what has been reported in a systematic review among children and adolescents.^7^ SMU is sometimes linked to emotional responses like stress, anxiety, or low self-esteem,^17,28^ which can lead to emotional eating. Social media food influencers and trends often highlight sugary treats like desserts and sugary drinks,^7^ encouraging followers to try them, especially when presented as fun or trendy. Additionally, this phenomenon might be driven by the advertising component on social media platforms. As suggested in a systematic review, food and beverage companies often use social media platforms to target the adolescent population, marketing their unhealthy products such as sweets and sugary drinks.^34^ This digital marketing strategy may play a role in shaping the dietary choices of adolescents.

The negative association between problematic and excessive SMU and habitual breakfast intake observed in this study was supported by previous studies in children and adolescents.^21,35^ This relationship between SMU and skipping breakfast is not limited to social media, as it has also been observed with other forms of screen-based behaviour, such as general screen time,^36^ playing electronic games,^37^ and television viewing.^38^ There are several potential explanations for this phenomenon. One possibility is that the time spent on SMU displaces the time available for having breakfast.^39^ SMU, particularly in the morning, may compete with the time needed to prepare and have a breakfast, leading adolescents to skip this important meal. In addition, the late sleep patterns (late sleep onset, late wakeup times) associated with the SMU^40^ may also disrupt regular routine, and ultimately result in the omission of breakfast.

Sex differences presented in fruits, vegetables and breakfast intake. Specifically, girls were less likely to intake fruits, vegetables, and breakfast than boys across SMU (both problematic and excessive use). This pattern suggests that SMU can affect certain dietary behaviours more than others, and these effects may differ by sex. One potential explanation could be that girls tend to use social media primarily for social interaction, emotional support, and maintaining relationships.^41,42^ This emotional reliance on social media may lead to dependency and distract them from self-care activities, such as eating balanced meals or breakfast. In contrast, boys are more likely to use social media for general information-seeking purposes,^42^ which is less emotionally consuming and less likely to interfere with daily routines like meal consumption. Additionally, girls are more prone to skipping breakfast,^43,44^ which may further exacerbate the disparity in dietary habits. Future research should address the unique challenges girls face in balancing SMU with healthy eating habits.

Emerging evidence highlights the critical role of nutrition knowledge and literacy in shaping adolescents’ dietary behaviours,^45^ particularly as they view food-related content on social media platforms. Our findings suggest that nutrition literacy may differentially influence problematic and excessive SMU, with distinct dietary outcomes. Both problematic and excessive SMU were associated with lower breakfast intake and higher consumption of sweets and sugary foods, likely due to shared behavioural drivers such as convenience-seeking and limited nutrition literacy. Breakfast is often time-consuming to prepare and frequently skipped due to lack of time and unavailability of food,^46^ while easily accessible sweets and sugary snacks align with adolescents’ prioritisation of immediate gratification.^47^ However, problematic SMU was uniquely linked to lower fruit and vegetable intake, whereas excessive SMU correlated with higher fruit and vegetable consumption. The detrimental impact of problematic SMU on fruit and vegetable intake may stem from its compulsive nature. Adolescents exhibiting problematic SMU may have impaired ability to critically evaluate conflicting food-related content (e.g., snack promotions vs. healthy eating advice), particularly when combined with low nutrition literacy.^48,49^ Conversely, adolescents who engage heavily but non-problematically with social media may encounter algorithm-driven recommendations for healthy recipes, plant-based diets, or peer-shared meal ideas, which could normalise and encourage fruit/vegetable consumption.^31^ These adolescents may mitigate the feelings of guilt associated with increased consumption of sweets and missed breakfasts by incorporating a greater variety of fruits and vegetables, guided by their growing nutritional knowledge.^50^ These diverging outcomes highlight that social media impacts nutritional knowledge and literacy in both positive and negative ways, potentially leading to healthy or unhealthy dietary behaviours. Therefore, understanding the mediating role of nutrition literacy should be a key focus moving forward.

This study shows strong connections between problematic and excessive SMU and poor eating habits in teenagers, which is important for public health. The main problem is how platforms create and share content using filters and AI tools. Social media companies could respond to user concerns by using AI to promote healthier choices, like sharing information about dietary guidelines and exercise. They could also encourage outdoor activities and local fresh food options instead of fast-food chains. These changes could improve both physical and mental health. Regulatory agencies might set rules for these platforms about food product advertising, especially to vulnerable groups like children. These regulations would aim to balance user freedom with public health goals, creating a safer online space.

To the best of our knowledge, this is the first study to examine the relationships between excessive and problematic SMU and dietary behaviours in 222,865 adolescents, using representative samples from 41 countries. Reliable and validated questionnaires were used to measure SMU and dietary behaviour. However, the study has some limitations. First, the use of self-reported data may be subject to various biases including recall bias. Future studies with more comprehensive (e.g., device based) measures are needed to more accurately explore these relationships. Second, the arbitrarily categorisation of behaviours may not best represent the true association estimates. Third, the cross-sectional nature of the study precludes the ability to infer causal directionality between the variables of interest. The findings of the present study challenge the traditional, simplistic view of a uniformly negative relationship between SMU and dietary behaviours. The potential differences between various SMU types should be considered when examining the relationships with health behaviours.

Decades of research on social media’s impact on users indicate that a new approach is necessary. Instead of a uniform method, it is important to recognise the complex ways social media relates to diet and health. Future research should focus on the purpose, content, context, and duration of SMU to uncover these relationships, aiding in the development of strategies to promote healthy behaviours among adolescents in the digital age. The US Office of the Surgeon General (2023) emphasized the need for social media companies to provide real-time data and work with independent researchers to assess the risks and benefits of social media.^51^ Developing methods to track user responses to specific content over time, rather than relying solely on one-time surveys, will help position social media as a force for good and encourage collaborations that foster well-being, such as promoting healthy eating behaviours.

## Conclusions

The findings indicate that adolescents reporting either problematic or excessive SMU patterns had higher chance to have an overall poor dietary intake. Problematic SMU was associated with lower intake of fruits, vegetables, and breakfast regardless of sex. In contrast, excessive SMU was associated with higher intake of fruits and vegetables. Both problematic and excessive SMU were associated with daily consumption of sweets and sugary drinks. The associations observed underscore the complex interplay between different SMU and multifaceted aspects of dietary behaviours. A new research approach is proposed that involves working with social media companies to obtain real-time data. Additionally, it is important to combine device-based evaluations of SMU, considering both content and context, with a thorough analysis of usual dietary intake to gain insights into the mechanisms affecting these varying relationships.

## Supplementary information

Appendix

## Data Availability

This study utilised publicly available data from the Health Behaviour in School-aged Children (HBSC) study, accessible at https://hbsc.org/data/.
