# Supplementary material for: Bytes and bites: social media use and dietary behaviours among adolescents across 41 countries
Source: Pediatr Res. 2025 Apr 7;98(6):2101–8. doi: 10.1038/s41390-025-04030-z (PMC12811122; doi:10.1038/s41390-025-04030-z)
Supplement: Supplementary file 1 — Appendix [file 41390_2025_4030_MOESM1_ESM.pdf]

Appendix – A

Table A1 Country estimates[§] of ***problematic*** social media use (SMU) with good dietary intake (poor as reference) in adolescent ***girls*** from 41 countries, HBSC 2017/2018

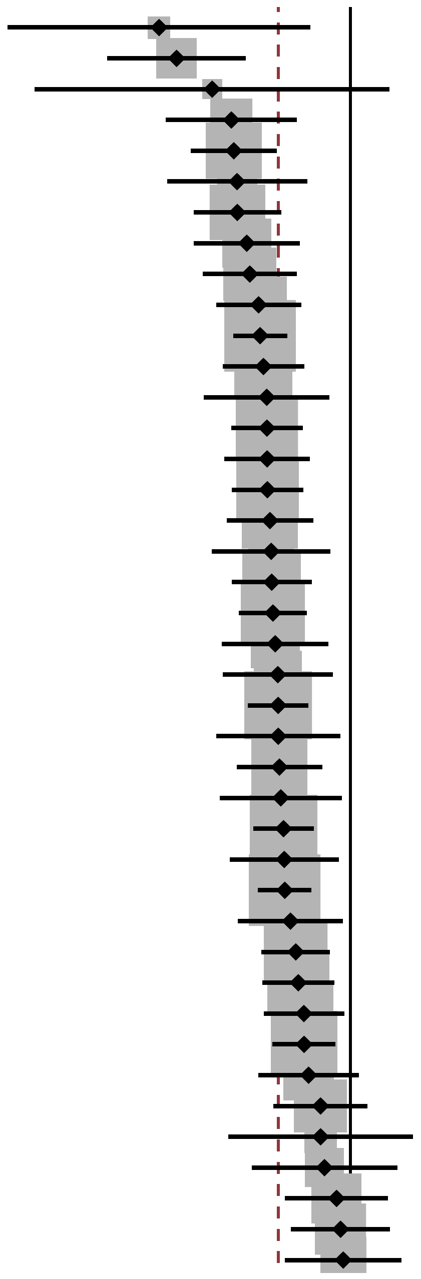

Country	OR (95% CI)
Israel	0.08 (0.01, 0.59)
Azerbaijan	0.10 (0.04, 0.25)
England	0.16 (0.02, 1.68)
Austria	0.21 (0.09, 0.49)
Ukraine	0.21 (0.12, 0.38)
Hungary	0.22 (0.09, 0.56)
Iceland	0.22 (0.13, 0.40)
Turkey	0.25 (0.13, 0.51)
Romania	0.26 (0.14, 0.49)
Macedonia	0.30 (0.17, 0.52)
Canada	0.30 (0.21, 0.43)
Greece	0.32 (0.18, 0.54)
Scotland	0.33 (0.14, 0.76)
Spain	0.33 (0.21, 0.53)
Lithuania	0.33 (0.19, 0.59)
Portugal	0.33 (0.21, 0.53)
Slovenia	0.34 (0.19, 0.61)
Denmark	0.35 (0.16, 0.77)
Republic of Moldova	0.35 (0.21, 0.60)
Wales	0.36 (0.23, 0.56)
Albania	0.37 (0.18, 0.75)
Latvia	0.38 (0.18, 0.79)
France	0.38 (0.26, 0.57)
Malta	0.38 (0.17, 0.87)
Estonia	0.39 (0.22, 0.69)
Ireland	0.40 (0.18, 0.89)
Belgium (French)	0.41 (0.28, 0.62)
Russia	0.42 (0.20, 0.86)
Czech Republic	0.42 (0.29, 0.60)
Luxembourg	0.45 (0.23, 0.91)
Italy	0.49 (0.31, 0.76)
Croatia	0.50 (0.31, 0.81)
Serbia	0.54 (0.32, 0.92)
Poland	0.54 (0.36, 0.82)
Germany	0.58 (0.30, 1.12)
Sweden	0.67 (0.36, 1.26)
Georgia	0.67 (0.20, 2.29)
Armenia	0.71 (0.27, 1.86)
Netherlands	0.83 (0.42, 1.65)
Norway	0.88 (0.46, 1.70)
Kazakhstan	0.91 (0.42, 1.97)

Table A2 Country estimates[§] of ***excessive*** social media use (SMU) with good dietary intake (poor as reference) in adolescent ***girls*** from 41 countries, HBSC 2017/2018

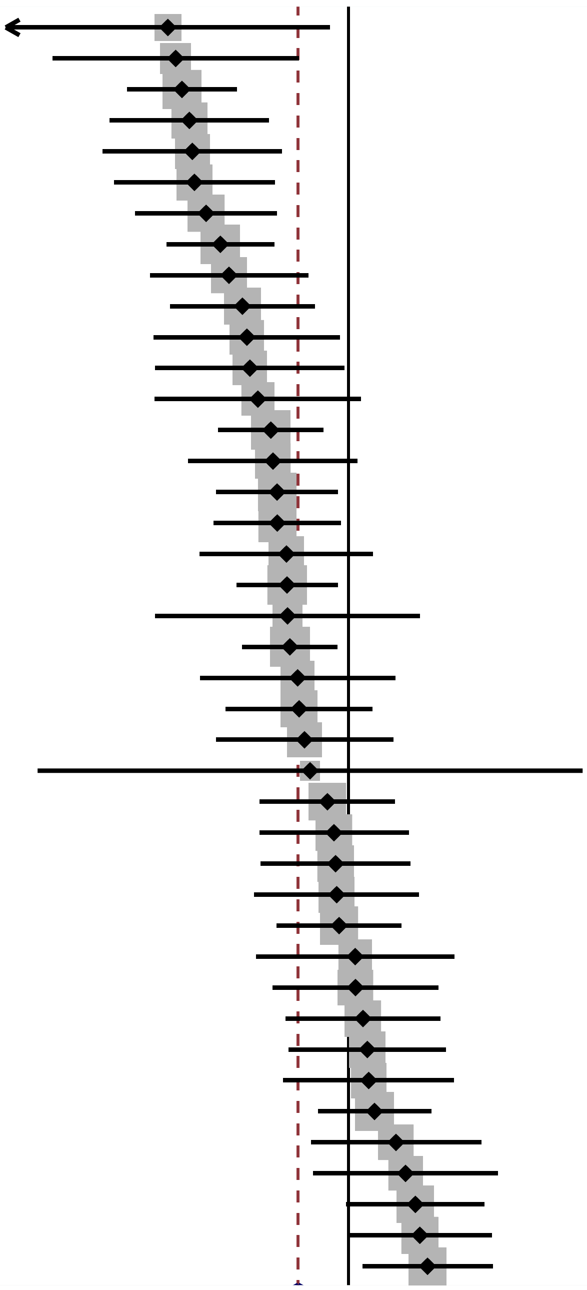

Country	OR (95% CI)
Ireland	0.51 (0.28, 0.93)
Scotland	0.53 (0.34, 0.83)
Portugal	0.54 (0.44, 0.66)
Wales	0.56 (0.42, 0.75)
Luxembourg	0.56 (0.40, 0.78)
Sweden	0.57 (0.42, 0.76)
Italy	0.59 (0.46, 0.77)
France	0.62 (0.51, 0.76)
Greece	0.64 (0.48, 0.86)
Spain	0.68 (0.52, 0.88)
Hungary	0.69 (0.49, 0.97)
Armenia	0.70 (0.49, 0.98)
Albania	0.72 (0.49, 1.05)
Belgium (French)	0.75 (0.62, 0.91)
Kazakhstan	0.76 (0.55, 1.03)
Iceland	0.77 (0.61, 0.96)
Slovenia	0.77 (0.61, 0.97)
Latvia	0.80 (0.58, 1.09)
Canada	0.80 (0.66, 0.96)
Malta	0.80 (0.49, 1.30)
Czech Republic	0.81 (0.68, 0.96)
Norway	0.83 (0.58, 1.19)
Croatia	0.83 (0.64, 1.09)
Romania	0.85 (0.61, 1.18)
England	0.87 (0.32, 2.37)
Macedonia	0.92 (0.72, 1.19)
Austria	0.95 (0.72, 1.25)
Serbia	0.95 (0.72, 1.26)
Netherlands	0.96 (0.71, 1.30)
Poland	0.97 (0.77, 1.21)
Georgia	1.02 (0.71, 1.48)
Denmark	1.03 (0.76, 1.39)
Germany	1.05 (0.79, 1.40)
Turkey	1.07 (0.80, 1.43)
Israel	1.08 (0.79, 1.47)
Ukraine	1.10 (0.89, 1.36)
Russia	1.19 (0.87, 1.63)
Azerbaijan	1.23 (0.88, 1.73)
Estonia	1.28 (0.99, 1.65)
Lithuania	1.30 (1.00, 1.69)
Republic of Moldova	1.34 (1.05, 1.70)

Table A3 Country estimates[§] of ***problematic*** social media use (SMU) with good dietary intake (poor as reference) in adolescent ***boys*** from 41 countries, HBSC 2017/2018

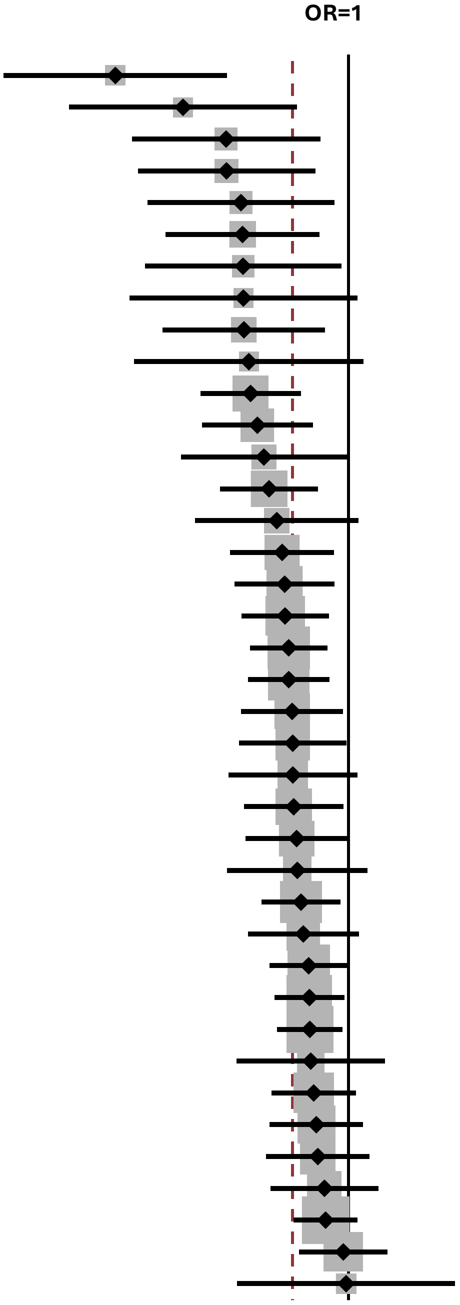

Country	OR (95% CI)
Azerbaijan	0.05 (0.01, 0.21)
Serbia	0.12 (0.03, 0.52)
Malta	0.21 (0.06, 0.70)
Ireland	0.21 (0.07, 0.66)
Germany	0.26 (0.08, 0.84)
Sweden	0.26 (0.10, 0.69)
Scotland	0.26 (0.08, 0.91)
Hungary	0.26 (0.06, 1.12)
Denmark	0.27 (0.10, 0.74)
Netherlands	0.28 (0.07, 1.21)
France	0.29 (0.15, 0.55)
Estonia	0.32 (0.16, 0.64)
Kazakhstan	0.34 (0.12, 0.98)
Ukraine	0.37 (0.20, 0.68)
Armenia	0.40 (0.14, 1.14)
Romania	0.43 (0.22, 0.83)
Macedonia	0.45 (0.24, 0.84)
Belgium (French)	0.45 (0.26, 0.78)
Wales	0.47 (0.29, 0.77)
Republic of Moldova	0.47 (0.28, 0.79)
Lithuania	0.49 (0.26, 0.93)
Poland	0.49 (0.25, 0.98)
Albania	0.49 (0.22, 1.12)
Slovenia	0.50 (0.27, 0.94)
Greece	0.52 (0.27, 0.99)
Austria	0.52 (0.22, 1.27)
Iceland	0.55 (0.33, 0.91)
Russia	0.56 (0.28, 1.14)
Portugal	0.60 (0.37, 0.99)
Czech Republic	0.61 (0.39, 0.95)
Canada	0.61 (0.40, 0.93)
Israel	0.62 (0.24, 1.59)
Turkey	0.64 (0.38, 1.10)
Croatia	0.67 (0.37, 1.20)
Norway	0.68 (0.35, 1.31)
Luxembourg	0.74 (0.37, 1.46)
Spain	0.75 (0.50, 1.12)
Italy	0.94 (0.54, 1.64)
England	0.97 (0.24, 3.86)

N=39 countries (without Georgia & Latvia)

Table A4 Country estimates[§] of ***excessive*** social media use (SMU) with good dietary intake (poor as reference) in adolescent ***boys*** from 41 countries, HBSC 2017/2018

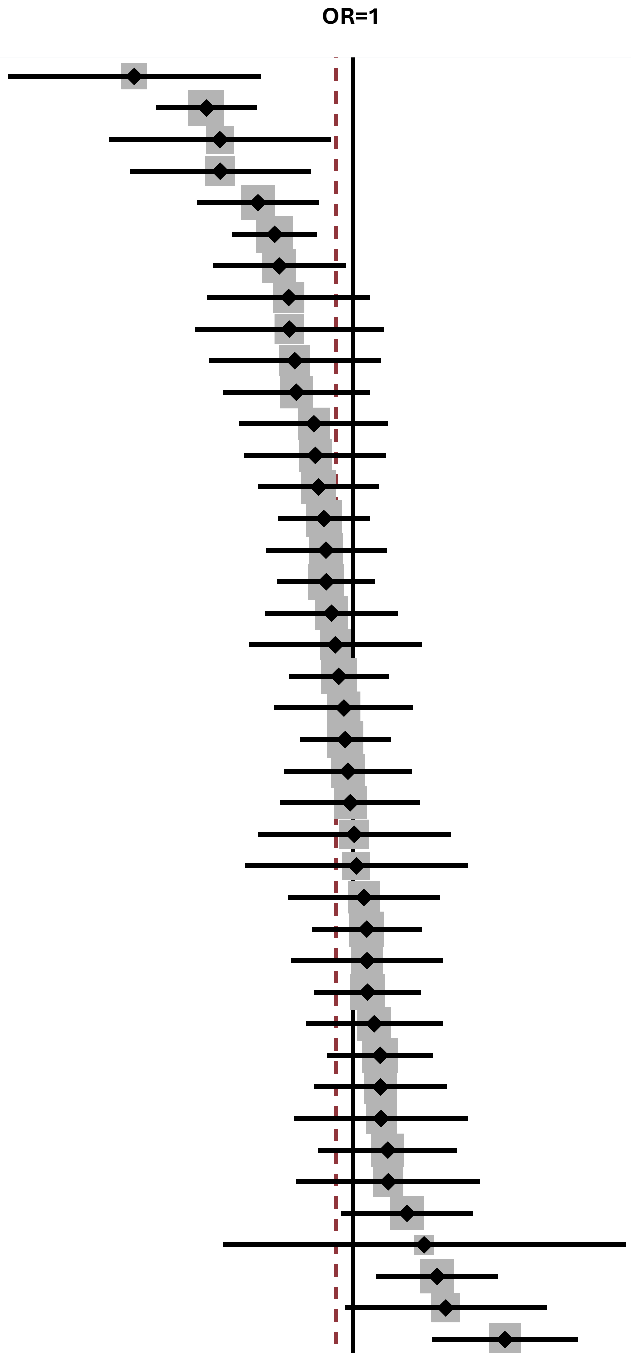

Country	OR (95% CI)
Ireland	0.36 (0.20, 0.65)
France	0.50 (0.40, 0.64)
Scotland	0.54 (0.32, 0.90)
Netherlands	0.54 (0.35, 0.82)
Wales	0.64 (0.48, 0.85)
Canada	0.69 (0.57, 0.85)
Sweden	0.71 (0.52, 0.96)
Norway	0.74 (0.51, 1.08)
Hungary	0.74 (0.48, 1.15)
Germany	0.76 (0.51, 1.14)
Austria	0.77 (0.54, 1.08)
Croatia	0.83 (0.59, 1.18)
Kazakhstan	0.84 (0.60, 1.17)
Macedonia	0.85 (0.64, 1.13)
Belgium (French)	0.87 (0.70, 1.08)
Poland	0.88 (0.66, 1.17)
Portugal	0.88 (0.70, 1.11)
Denmark	0.90 (0.66, 1.23)
Latvia	0.92 (0.61, 1.38)
Iceland	0.93 (0.74, 1.18)
Italy	0.96 (0.69, 1.32)
Czech Republic	0.96 (0.78, 1.19)
Spain	0.98 (0.72, 1.32)
Romania	0.99 (0.71, 1.37)
Armenia	1.01 (0.64, 1.58)
Malta	1.02 (0.60, 1.71)
Luxembourg	1.05 (0.74, 1.50)
Ukraine	1.07 (0.82, 1.38)
Israel	1.07 (0.75, 1.52)
Slovenia	1.07 (0.83, 1.38)
Greece	1.10 (0.80, 1.52)
Republic of Moldova	1.14 (0.89, 1.45)
Lithuania	1.14 (0.83, 1.55)
Georgia	1.14 (0.76, 1.71)
Serbia	1.18 (0.85, 1.63)
Azerbaijan	1.18 (0.77, 1.81)
Turkey	1.29 (0.95, 1.75)
England	1.39 (0.54, 3.58)
Estonia	1.48 (1.11, 1.97)
Albania	1.54 (0.96, 2.48)
Russia	2.03 (1.44, 2.87)
